# Exploring factors associated with bulk tank milk urea nitrogen in Central Thailand

**DOI:** 10.14202/vetworld.2018.642-648

**Published:** 2018-05-18

**Authors:** Suppada Kananub, Wassana Jawjaroensri, John VanLeeuwen, Henrik Stryhn, Pipat Arunvipas

**Affiliations:** 1 Department of Large Animals and Wildlife Clinical Sciences, Faculty of Veterinary Medicine, Kasetsart University, Bangkok, Thailand; 2 Laboratory Unit, Kasetsart University Veterinary Teaching Hospital, Nong Pho, Ratchaburi Province, Thailand; 3 Department of Health Management, Atlantic Veterinary College, University of Prince Edward Island, Charlottetown, Canada

**Keywords:** bulk tank milk urea nitrogen, farm level, non-nutritional factor, Thailand

## Abstract

**Aim:**

The study was to determine seasonal fluctuations and non-nutritional factors associated with bulk tank milk urea nitrogen(BTMUN).

**Materials and Methods:**

A total of 58,364 BTM testing records were collected from 2364 farms in Central Thailand during September 2014-August 2015. Using square root BTMUN as the outcome, other milk components, farm effect, and sampling time were analyzed by univariable repeated measures linear regression, and significant variables were included in multivariable repeated measures linear regression.

**Results:**

The average BTMUN (standard deviation) was 4.71 (±1.16) mmol/L. In the final model, BTM fat and protein percentages were associated with BTMUN as quadratic and cubic polynomials, respectively. BTM lactose percentage and the natural logarithm of somatic cell counts were negatively linearly associated with BTMUN. At the farm level, the BTM lactose association was negatively linear; herd BTMUN decreased following an increase of herd lactose average, and BTM lactose slopes were quite different among farms as well. Sampling time had the highest potency for the estimation of BTMUN over time, with lows and highs occurring in August and October, respectively. The variation in test level BTMUN was decreased by 18.6% compared to the null model, and 6% of the variance could be explained at the farm level.

**Conclusion:**

The results clarify seasonal variation in BTMUN and the relationships among other BTM constituents and BTMUN, which may be useful for understanding how to manage lactating dairy cattle better to keep BTM constituents within normal ranges.

## Introduction

The use of bulk tank milk(BTM) data has become a common tool for the evaluation of farm practices for the dairy industry, as it is economically efficient and convenient [1]. BTM data can be used to reflect overall health status, udder health, and nutritional balance between energy and protein [2,3]. Since milk urea nitrogen(MUN) corresponds well with blood urea nitrogen [4], over- and under-feeding of protein in a herd could show up in fluctuations of BTM urea nitrogen (BTMUN) [5]. Imbalances in protein and energy feeding can lead to excess protein in the rumen, and the nitrogen from the protein that is not utilized by ruminal microbes is converted to ammonia which leads to some impacts. Ammonia diffuses through the rumen wall into the blood, which goes to the liver and is detoxified into urea [2,6]. Urea circulates into blood and is eliminated through three pathways: Recycling by saliva, secretion through milk, and excretion in urine [5]. High urea causes uterine pH to change, and this is toxic to sperm, ova, and embryos and corresponds to impaired reproduction [7-9]. In addition, urea formation in the detoxification process consumes energy that will indirectly impair fertility [3,8].

Due to the relationship of MUN to reproductive problems, monitoring and control of MUN, at the farm level, could support nutritional measures to improve reproductive performance [7]. However, variations in BTMUN also occur from some non-nutritional factors that can vary from context to context [10,11], including factors such as milk yield, season, and days in milk [12,13].In Thailand, BTM testing includes percentages of fat, protein, lactose, and total solids (TS), as well as somatic cell counts (SCCs) and BTMUN. There are numerous studies of milk composition in Thailand, but few researchers have paid specific attention to BTMUN, or examined variability throughout seasons of the year.

Therefore, the objectives of our study were to describe the BTM compositions in Thai dairy farming over a year and to identify factors associated with BTMUN from the available data routinely collected in Thailand.

## Materials and Methods

### Ethical approval

This study obtained the permission of the Animal Ethics Committee by Laboratory Animals, Veterinary Technology, Kasetsart University (ACKU 59-VET-031).

### Sample description

All BTM testing records during September 2014-August 2015 were requested from a dairy cooperative in Central Thailand. Routine test components, herd identification, and a number of milking cows were the basic information recorded by the dairy cooperative for each BTM sample. Routine BTM testing was typically done 3 times a month; however, due to logistical difficulties, some months had only 1 or 2 samples taken from some farms. Milk samples were measured for percentages of fat, protein, lactose, and TS, as well as MUN concentration by Fourier transform infrared, while flow cytometry technology was utilized for detecting the number of somatic cells.

The data started with 62,159 records from 2,777 farms. To reduce possible biases, exclusion criteria consisted of: (1) Observations for farms without a record of herd size; (2) duplicated data from the same farm and date; and (3) farms sampled fewer than 5 times over the study period. Finally, data analyses were performed with 58,364 observations from 2364 herds.

### Data analysis

The descriptive statistical analysis was used to illustrate the central tendency and distribution of BTM components. To explore the factors associated with BTMUN, the following independent variables were considered: BTM percentage of milk fat, protein, lactose, and TS, BTM SCC, milking herd size, a period of month, and sampling time. Period of the month included the following categories: Early (days 1-10), mid (days 11-20), and late (days 21 to month end). The sampling time for each farm record was denoted with “1” corresponding to the first record in the sampling period and “36” as the last record in the sampling period. With high skewness, BTM SCC was reported and analyzed in the form of a natural logarithm transformation. As all data were at the BTM level, we will no longer use BTM when referring to the milk constituents, but we will continue to use BTMUN as a reminder of the BTM level of the outcome.

The analytical statistics were based on a mixed linear model of BTMUN (the outcome) that considered herd identification number as a random farm effect to adjust for clustering of repeated measures within farms. An exponential within-farm correlation structure was used in the model to adjust for strong similarities between contiguous samples among the repeated measures because the gaps between sampling times were unequal. A Box-Cox analysis was utilized for BTMUN to determine the best transformation to ensure that the model residuals approximately had a normal distribution. Continuous independent variables were centered by subtracting the mean so that the association would explain variation around the average. We used Lowess smoothing plots graphed to visualize relationships between continuous variables and BTMUN, and polynomial regression was also used for independent continuous variables to determine if its relationship with BTMUN was curvilinear. To evaluate model assumptions, homoscedasticity was checked for constant variance at the test level, and normality of residuals was tested, whereas at the farm level, predicted random effects were tested for normality.

Individual independent variables were initially screened for associations with BTMUN, with a cutoff of p<0.20, and eligible variables were used to build the multivariable model using a backward manual stepwise process. p<0.05 was used to examine the significant variables as well as interaction in the final model. To determine confounding effects, variables not retained in the model were forced back into the model and coefficients of model variables were observed for changes >20%, indicating a potential of confounding. Any polynomial variables in the final model were illustrated with a graph to clarify understanding. Unless noted otherwise, predictions were done with variables set at their average value.

The variation of the coefficients between farms was further explored by contextual effects and random slopes [14]. The herd means and random slopes for effects that were individually significant were offered to the model. Even though several variables showed statistically significant random slopes, for ease of interpretation, the final model would retain only one variable with a random slope (i.e., the random slope with the largest improvement in fit).

## Results

The number of observations was initially 62,159 records from 2,777 farms. For 3,148 records, there were no data of herd size, so they were excluded from the study. To eliminate redundant data, 527 duplicate records were removed, and 120 records from farms tested <5 times over the year were considered to have insufficient data and were not included in the analyses. As a result of these exclusion criteria, data analyses were performed on a total of 58,364 records from 2364 farms that had complete recorded data. The number of records per farm varied from 5 to 28. Minimal and maximal herd sizes were 1 and 100 cows, respectively, and 50% of farms had fewer than 10 milking cows. Descriptive statistics of BTM compositions, the natural logarithm of SCCs (LNSCC), and BTMUN are presented in Table-1.

**Table-1:** Descriptive statistics of milk constituents for 58,364 bulk tank samples from 2,364 farms in Thailand from September 2014 to August 2015.

Milk constituents	Mean±SD	Range	Percentiles (%)	

			25	75
Fat (%)	3.86±0.46	0.55-8.06	3.56	4.13
Protein (%)	3.21±0.24	1.95-5.19	3.05	3.35
Lactose (%)	4.62±0.15	2.99-5.24	4.54	4.72
TS (%)	12.39±0.62	7.91-17.99	11.99	12.75
MUN (mmol/L)	4.71±1.16	0.71-12.04	3.93	5.64
LNSCC	13.07±0.88	9.55-16.98	12.48	13.66

TS=Total solids, MUN=Milk urea nitrogen, LNSCC=Natural logarithm of somatic cell counts

There were very wide ranges of fat, TS, MUN, and LNSCC on the BTM, whereas the gaps between 25^th^ and 75^th^ percentiles were not broad. The geometric mean SCC, after back-transformation, was 489x10^3^ cells/ml, with values ranging from 15x10^3^ cells/ml to 24,155x10^3^ cells/ml. Box-Cox analysis suggested a square root transformation of the BTMUN outcome for the analytical statistics. Univariable analyses indicated that milk fat, protein, lactose, TS, and LNSCC had significant associations with square root BTMUN. Milk fat and protein were strongly correlated with TS, defined by Pearson correlations of 0.9 and 0.8, respectively. Furthermore, those factors had stronger predictive ability than TS, and therefore, TS was omitted from the modeling process. Period of the month was excluded from the modeling process as well because of collinearity with sampling time as a categorical variable.

After the fixed-effect model had been completed, herd-level random effect slopes for the milk constituents were added to the model to improve the model’s fit and interpretation. Each milk constituent was individually interpreted, with the largest improvement in model fit (in terms of the Akaike’s information criterion) achieved for lactose (Table-2). To evaluate confounding effects, herd size was forced back into the model analysis, but the change of the coefficients was <20%, and therefore, herd size was not considered to be a confounding variable. Table-3 expresses the significant fixed and random effects that were associated with the square root of BTMUN in the final model.

**Table-2:** LL and AIC informing the fit of statistical models (fixed effect model which included different individual random slopes) for square root BTMUN for 58,364 samples from 2,364 Thai farms in 2014-2015.

Models	LL	AIC
No random slope	−16863.78	33819.56
Fat	−16404.91	32911.82
Protein	−16417.08	32944.17
Lactose	−16323.21	32742.42
LNSCC	−16715.17	33526.35

LL=Log likelihood, AIC=Akaike’s information criteria, BTMUN=Bulk tank milk urea nitrogen, LNSCC=Natural logarithm of somatic cell counts

The relationships of milk fat and protein to BTMUN were curvilinear, with milk fat related by a quadratic association and milk protein by a cubic association (Table-3). Figure-1 presents milk fat and milk protein associations, estimated based on the third sampling of October, adjusting for time effects. Figure-1(a) presented the peak of the estimated BTMUN around 3% milk fat, and the BTMUN level was lower at lower and higher fat percentages, with variability increasing at low and high milk fat. For milk protein, the MUN was lower and higher when milk protein was below 3.0% and above 4.0%, respectively, but changed only slightly between milk proteins of 3.0 and 4.0%, with BTMUN variability again increasing at low and high milk protein (Figure-1(b)).

**Table-3:** Final multivariate mixed model representing variables associated with square root BTMUN, and variation at each level for 58,364 samples from 2,364 Thai farms in 2014-2015.

Factor	Type	Estimate	SE	95% CI	p value
Fixed effects					
Fat	Linear	−0.066	0.005	−0.075-−0.057	<0.001
	Quadratic	−0.040	0.004	−0.048-−0.032	<0.001
Protein	Linear	−0.008	0.011	−0.028-0.013	0.466
	Quadratic	−0.065	0.022	−0.109-−0.021	0.004
	Cubic	0.083	0.025	0.035-0.132	0.001
F_lactose^a^	Linear	0.292	0.058	0.178-0.406	<0.001
Lactose	Linear	−0.569	0.021	−0.611-−0.527	<0.001
LNSCC	Linear	−0.031	0.002	−0.036-−0.027	<0.001
Time (1^st^ time is ref)	Categorical	-	-	-	<0.001
Constant	-	2.371	0.269	1.844-2.898	<0.001

**Factor**	**Parameter**	**Estimate**	**SE**	**95% CI**	**p value**

Random effects					
Farm	Variance	0.078	0.003	0.073-0.083	-
Lactose	Variance	0.418	0.025	0.372-0.469	-
	Covariance	0.045	0.006	0.034-0.057	-
Test	Variance	0.096	0.001	0.095-0.097	-
	Rho (correlation coefficient)	0.869	0.002	0.866-0.873	-

a farm average of percentage lactose, BTMUN=Bulk tank milk urea nitrogen, SE=Standard error, CI=Confidence intervals, LNSCC=Natural logarithm of somatic cell counts

**Fiure-1:**
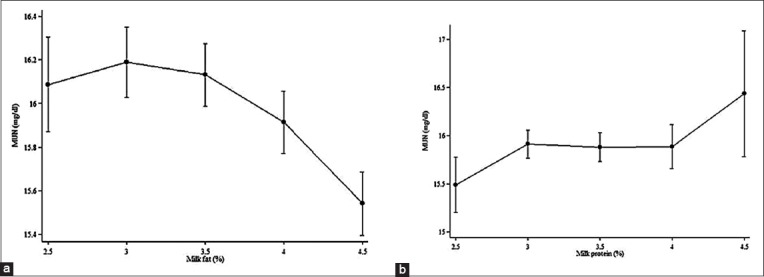
Prediction of bulk tank milk urea nitrogen concentrations estimated by percentage of milk fat (a) and protein (b), respectively (95% confidence interval of prediction presented by error bar) for 58,364 samples from 2,364 Thai farms in 2014-2015.

Lactose, LNSCC, and herd average of lactose were linearly associated with BTMUN (Table-3). For an individual sample, each unit decrease of lactose from its mean was associated with an increase of 0.57 units in the square root BTMUN (95% confidence intervals [CI]: 0.53-0.61). However, for the significant herd-level contextual effect, a herd mean decrease in lactose of 1 unit was associated with a herd mean increase of square root BTMUN of 0.29 units. When LNSCC increased by 1 unit from the average, square root BTMUN was lower by 0.031 units (95% CI: 0.027-0.036) (Table-3).

**Figure-2:**
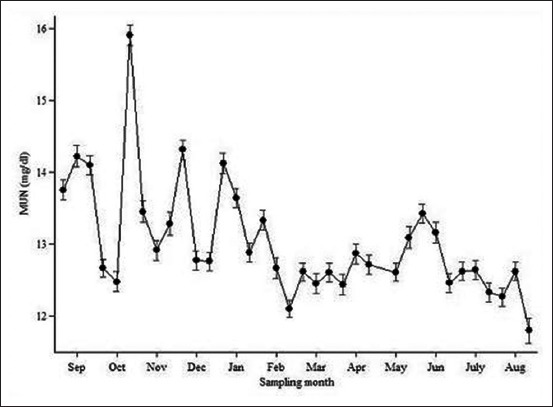
Prediction of bulk tank milk urea nitrogen estimated by sampling time (95% confidence intervals of prediction represented by error bars) for 58,364 samples from 2,364 Thai farms in 2014-2015.

Sampling time was also significantly associated with square root BTMUN (Table-3). The predictions of BTMUN fluctuated substantially, particularly in the first 6 months of the monitoring period (Figure-2). There seemed to be a pattern of high BTMUN concentration in the early part of the month, which went down in the middle and later parts of the month, especially in December, January, February, and June.

Considering the variation presented at the farm level, it accounted for approximately 45% of the unexplained variation in the model (Table-3). Additionally, the effect of lactose varied among farms, with 95% probability a herd-specific slope for lactose would be within nearly 2 times 0.65 (the herd lactose standard deviation calculated from the square root of the lactose variance at the herd level) of the average slope of −0.57, corresponding to a 95% CI range from −1.87 to 0.73.

Table-4 presents the estimated BTMUNs from the final model in the scenarios representing 25^th^ and 75^th^ percentiles of milk fat, protein, and lactose at the sampling time of the end of October and August when the highest and lowest predicted BTMUN values transpired, respectively. Accounting for milk fat, the BTMUN estimations decreased by only 1.8 to 2.2%. BTMUN only slightly varied with milk protein from 0.06 to 0.09% between Q1 and Q3. Milk lactose was the milk component that had the highest influence on the BTMUN outcome, leading to about 5% difference in the prediction. The greatest alteration in BTMUN, by 25% approximately, was found for the variable of time - August versus October.

**Table-4:** Prediction of BTMUN estimated by percent of milk fat, protein, lactose, and sampling time for 58,364 samples from 2,364 Thai farms in 2014-2015.

Milk fat	Milk protein	Milk lactose			

		4.60^a^		4.65^b^	
	
		October	August	October	August
3.56^a^	3.05^a^	16.49	12.30	15.67	11.60
	3.35^b^	16.48	12.29	15.66	11.59
4.13^b^	3.05	16.19	12.05	15.38	11.34
	3.35	16.18	12.04	15.37	11.34

a 25^th^ percentile of variable,

b 75^th^ percentile of variable, BTMUN=Bulk tank milk urea nitrogen

The null model was compared with the full model in Table-5, describing the variance explained by the parameters in this study. The variation was dropped by 18.6% from the null to the full model at the test level, while at the herd level, the variation was reduced by 6%. There were other factors (e.g., nutritional factors) associated with BTMUN which were not contained in this model due to lack of data.

**Table-5:** Variance of test level and herd level in the null model and full model for 58,364 samples from 2364 Thai farms in 2014.2015..

Models	Level of variance	

	Herd	Test
Null model^a^	0.083	0.118
Full model^b^	0.078 (6%)^c^	0.096 (18.6%)^c^

a Only intercept and herd random effect contained in model

b model with fixed and herd random effect

c percentage of variance explained by full model

BTMUN=Bulk tank milk urea nitrogen

## Discussion

This study demonstrates the substantial seasonal variation of BTMUN over the year and also helps inform the relationships between BTMUN and other BTM components in Thailand. This information could assist the dairy industry on how to manage farms to minimize the fluctuations in BTMUN out of the normal range.

### Descriptive statistics

Our average milk fat, protein, lactose, and TS results were slightly higher than other studies in Thailand, as reported by Sakhong [15] and Yeamkong et al. [16], likely because of the different study areas and time. Type and quality of roughages are dependent on farm management and season [17], and these factors directly impact the percentage of milk fat [18].

SCCs were lower than the studies by Jarassaeng et al. [11] and Tangjitwattanachai [19] but higher than those found by the Yeamkong et al. [16]. Typically, high BTSCC represents subclinical intramammary infections in cows on farms because cows usually have clinical mastitis less frequently than subclinical mastitis, and usually farmers would not mix milk affected by clinical mastitis into the milk they are selling. The Thai Agricultural Standard for BTSCC at the time of the study was <500×103 cells/ml for raw milk that was gathered into a milk production line [20]. The BTSCC among the study farms remained around the standard, even though there have been milk quality control programs implemented in the region in the past.

The BTMUN data reported previously in Thailand were collected in clinical trials [6,21], and therefore, there was no reference level from an observational study to compare our results in Thailand. Various cut points for normal and abnormal MUN concentrations have been mentioned; a MUN of 2.86-4.29 mmol/L is recommended as a normal range [5], and some studies looked at 5.71-6.43 mmol/L to categorize MUN as a high level [10,22]. Our BTMUN average, 4.71 mmol/L, was in between these two ranges, with approximately 20% of observations in the 5-5.71 mmol/L and 18% >5.71 mmol/L, demonstrating that some farms sometimes had high BTMUN.

### Analytical statistics

Milk fat was negatively associated with BTMUN, as found by Arunvipas *et al*. [12] and Cao *et al*. [13]. A higher percentage of milk fat could reflect a higher amount of energy that cows are getting from the feed, reducing the production of MUN [23]. Rumen microbes consume rumen energy and protein to form microbial protein. If the amounts of protein and energy are not balanced due to insufficient and/or unavailable energy, ammonia would diffuse through the rumen wall and be changed to urea in the liver, increasing MUN [2,18].

The non-linear association of milk protein to BTMUN that existed in this study differed from other studies [12,24] which found a negative linear association. Not only the amount of crude protein in the ration but also the kinds of protein fed (rumen degradable protein or rumen undegradable protein) likely contribute to the observed differences in BTMUN versus milk protein between studies [25,26]. Over- and under-feeding of protein relative to dietary energy could show up in fluctuations of BTMUN [5]. Degradable protein has a more apparent influence on the milk urea formation than non-degradable protein [8], and as such, different protein degradabilities and usages in each area could lead to dissimilar associations.

The negative linear relationship between lactose and MUN was also identified in previous studies [22,27,28], but a different finding was observed in Cao et al. [13], where a parabolic relationship between lactose and MUN concentration was found. Elsewhere, a higher milk yield corresponded to higher milk lactose, which subsequently resulted in diluting the MUN concentration [22,27]. Unfortunately, there were no available data on milk yield in our study. We did note that the influence of milk lactose existed not only as a main fixed effect for individual BTM samples on farms but also as a random effect for the herd mean lactose level, given the considerable differences in lactose slopes among herds. Further research should include milk yield to clarify this association, if possible.

Our findings revealed a negative linear association between BTMUN and LNSCC, which was similar to the study of Arunvipas et al. [12] but different from the study of Yoon et al. [29]. Two opposing response mechanisms to mastitis have been considered: (1) Bacterial release of urease in the udder that would degrade urea, having a negative effect on MUN; and (2) high permeability to MUN of infected udder tissue, in which urea would increasingly be released into the udder [13]. Due to these two mechanisms, positive or negative associations could happen, depending on which mechanisms have dominated the udder.

The association between sampling time and MUN has not been demonstrated in Thailand before. The results showed a significant time effect during the year, and some variability in the BTMUN results within the same month. Possible causes of the lower MUN during the year could be decreasing feed consumption and lowered quality of fodder [29,30]. Limitations in amount and quality of roughage often occur in dry seasons such that silage or agricultural residuals are used instead, especially in tropical countries [17]. In Thailand from November to April, the cool, dry season, there is often a fluctuation of fodders fed to cows due to the slow growth of grasses, causing a shift of the main roughage to rice straw [21]. As a result of a shortage of good quality roughage, the concentrate is often added in the feed ration, which could lead to dietary protein and energy imbalances, with increased variation and higher MUNs transpiring [21,30]. The quantity and continuity of precipitation in rainy seasons in Thailand can also change over the years, affecting the growth and quality of forages fed. Prices of feeds vary depending on availability, and therefore, a farmer’s nutrition decisions may rely on lower quality and quantities of feeds when prices are high [17] leading to variation of BTMUN in some months. Yoon *et al*. [29] also found lower BTMUN in the months mentioned to be a dry season (February to May) in Korea, corroborating our findings.

Although the study covers a large area of the dairy industry in Thailand, the data were obtained from only one source, so there is a possible limitation of representativeness of the study population for all of Thailand or elsewhere. Possible biases occurring in this study were as follows: (1) 413 farms were excluded at the beginning of model building because there were no records of herd size, and also herd size was typically estimated by the processing company employees for many farms, with this measurement error possibly contributing to it not being a significant variable in the model; (2) farms with <5 milk records over the study period were excluded from the analyses because their information was not considered to be reliable, possibly leading to a selection bias; (3) there were missing data points for all farms, for various logistical reasons, potentially leading to missing data bias if the reasons for them missing are related to both the outcome and the predictors investigated; (4) the associations observed may be peculiar to the weather conditions of the particular year being studied; and (5) the model did not include nutritional factors due to unavailable data, potentially leading to confounding bias.

Recommendations for further study of BTMUN in Thailand include obtaining data with fewer missing data and obtaining data for multiple years to determine if associations observed are consistent through weather conditions from multiple years. Coupling of these BTMUN data with more data on the farm, such as feeding management, would also be helpful to provide a complete understanding of factors associated with BTMUN.

## Conclusion

The average BTMUN for this population of dairy farms in Thailand during the study period was 4.71 mmol/L. A positive relationship with BTMUN was found for milk protein (curvilinear), while milk fat (curvilinear), lactose, and SCC had negative linear associations. Sampling time was also associated with BTMUN, with higher and more fluctuating MUNs during the first half of the year (dry winter months). The chief variation of BTMUN estimation was found among farms, emphasizing that strategies to control BTMUN fluctuations and aberrations should be directed primarily at the farm level. The results clarify seasonal variation in BTMUN and the relationships among other BTM constituents and BTMUN, which may be useful for understanding how to manage lactating dairy cattle better to keep milk constituents, especially BTMUN, within normal ranges.

## Authors’ Contributions

JV, HS, and PA are main supervisors, giving the suggestions for the analyses and interpretation, in addition to revising correctness of the manuscript. WJ was responsible for sample analyses. SK had the responsibilities for the sample collection, the data analyses, and the manuscript writing. All authors read and approved the final manuscript.
